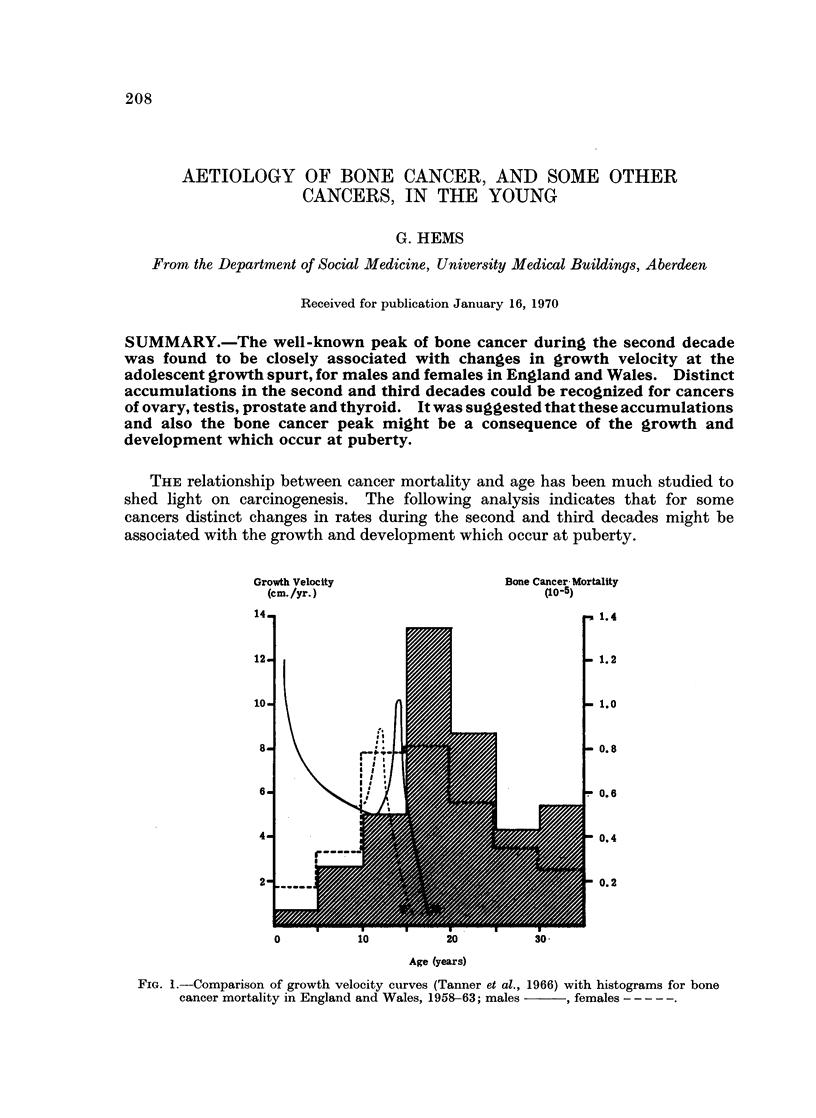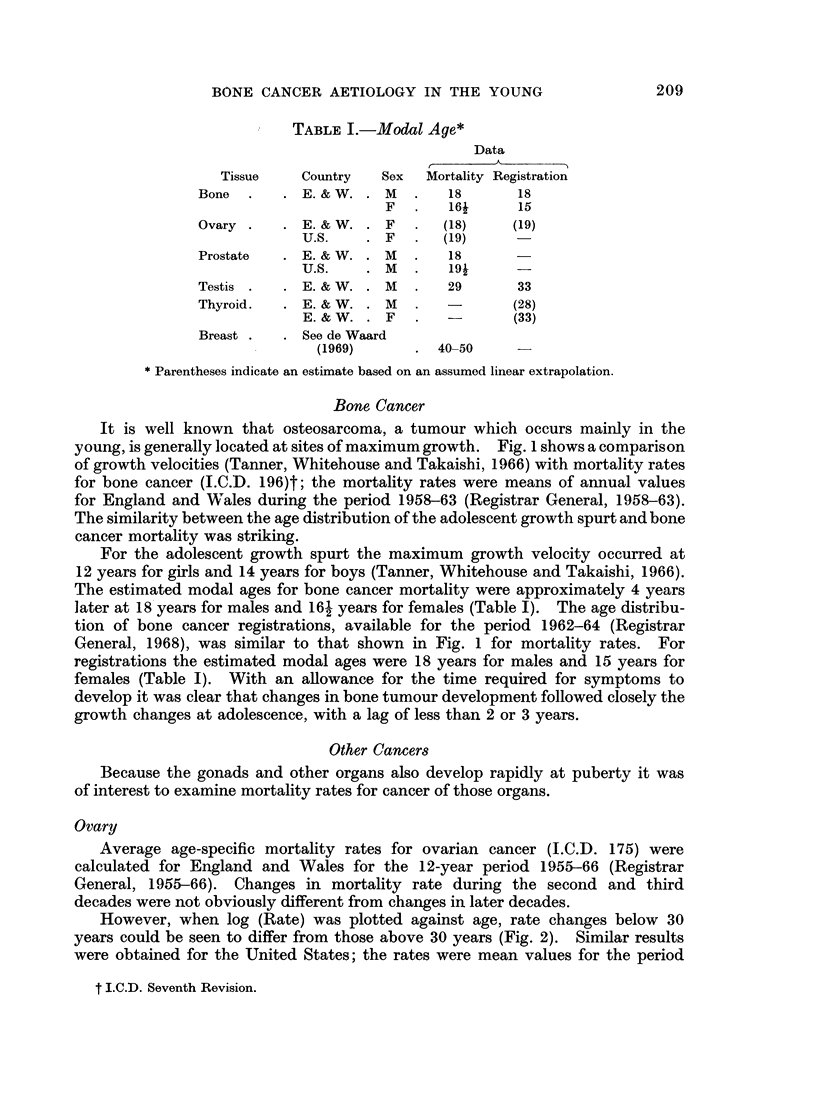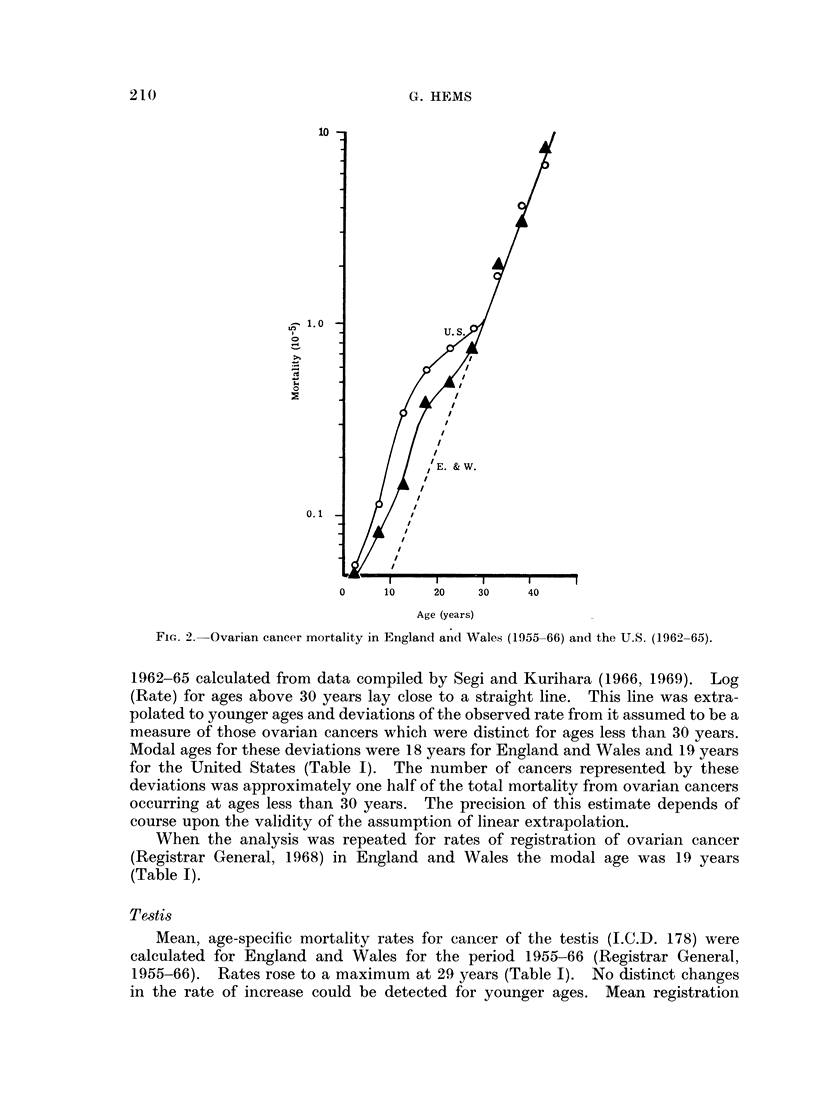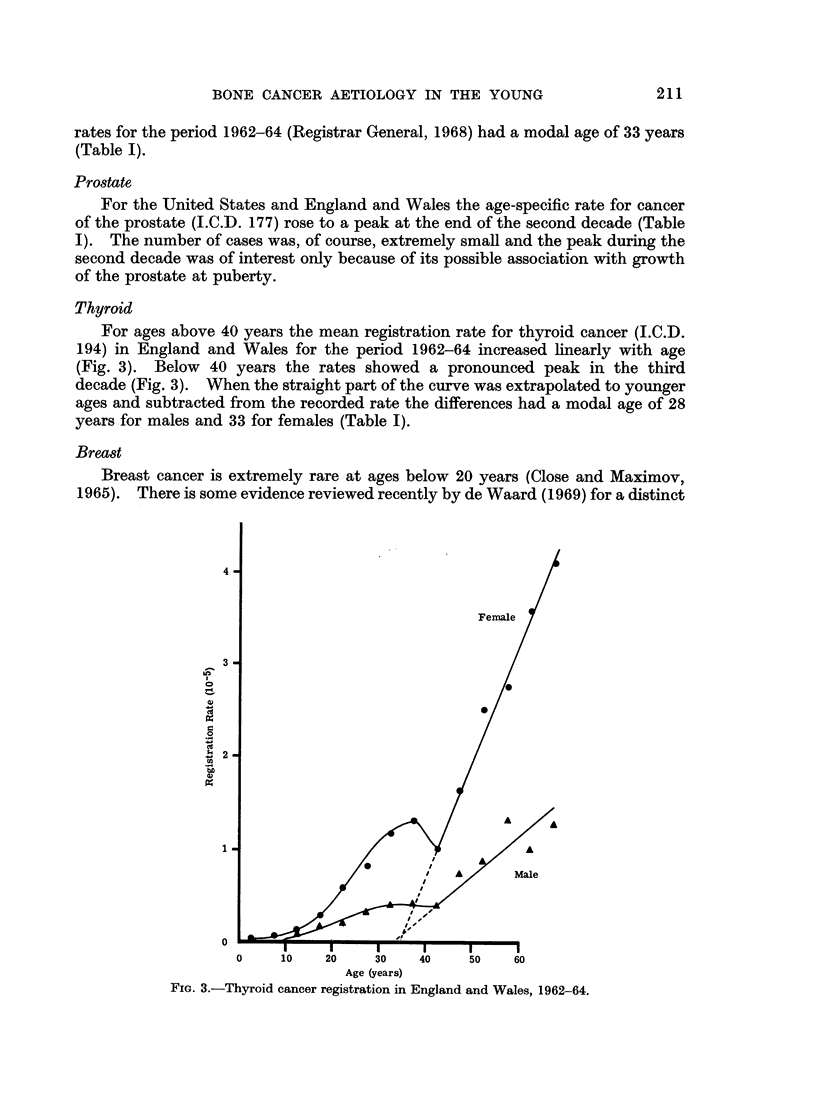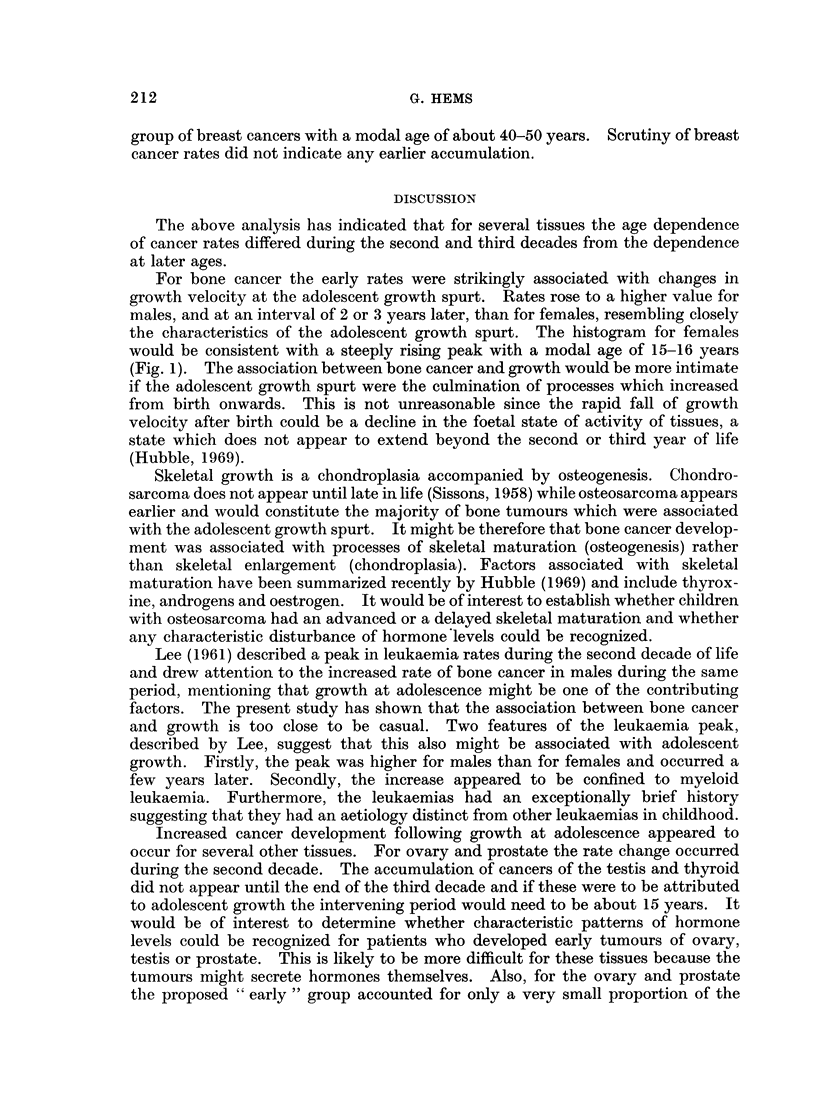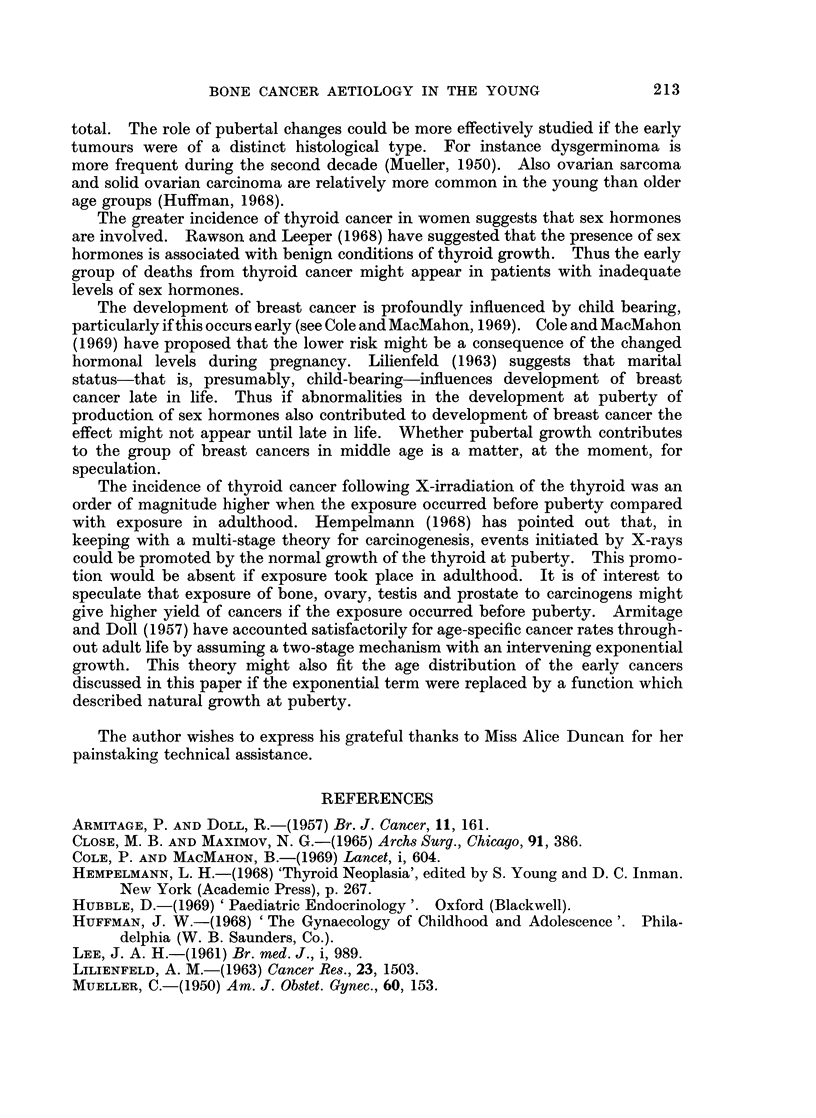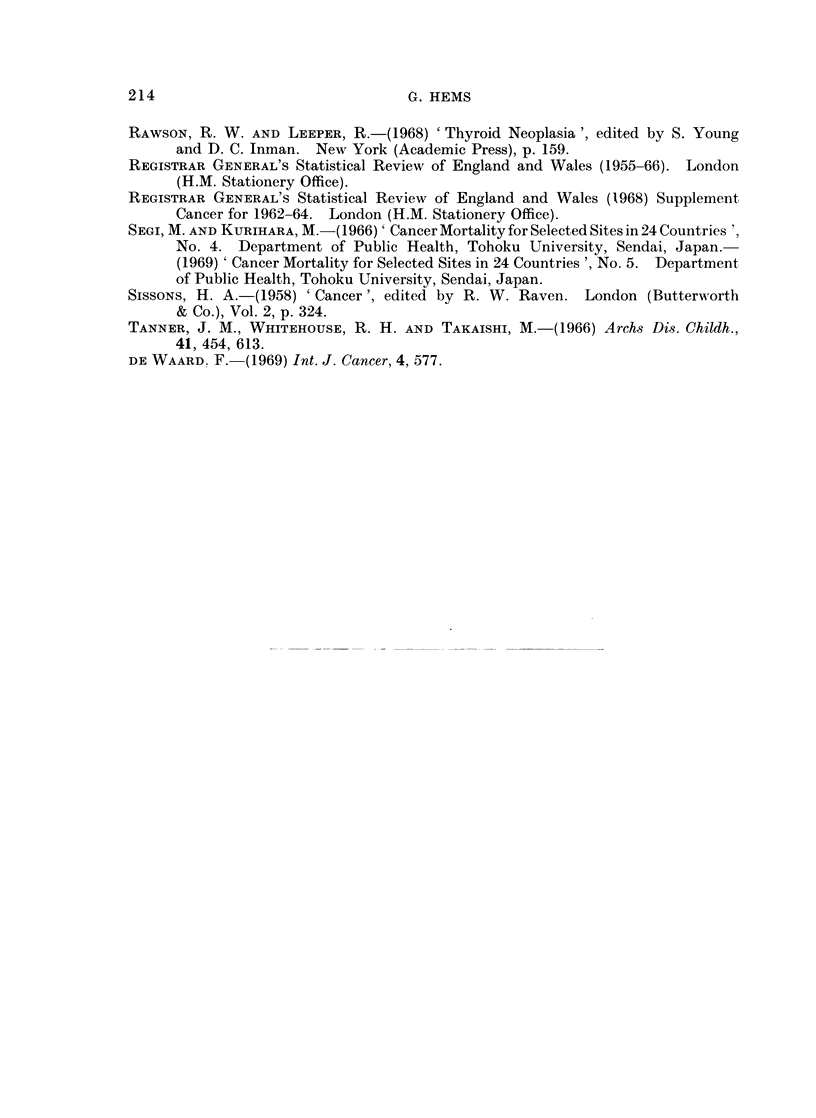# Aetiology of Bone Cancer and Some Other Cancers in the Young

**DOI:** 10.1038/bjc.1970.24

**Published:** 1970-06

**Authors:** G. Hems

## Abstract

The well-known peak of bone cancer during the second decade was found to be closely associated with changes in growth velocity at the adolescent growth spurt, for males and females in England and Wales. Distinct accumulations in the second and third decades could be recognized for cancers of ovary, testis, prostate and thyroid. It was suggested that these accumulations and also the bone cancer peak might be a consequence of the growth and development which occur at puberty.


### AETIOLOGY OF BONE CANCER, AND SOME OTHER CANCERS, IN THE YOUNG

G. HEMS

*From the Department of Social Medicine, University Medical Buildings, Aberdeen*

Received for publication January 16, 1970

**SUMMARY.—The well-known peak of bone cancer during the second decade was found to be closely associated with changes in growth velocity at the adolescent growth spurt, for males and females in England and Wales. Distinct accumulations in the second and third decades could be recognized for cancers of ovary, testis, prostate and thyroid. It was suggested that these accumulations and also the bone cancer peak might be a consequence of the growth and development which occur at puberty.**

THE relationship between cancer mortality and age has been much studied to shed light on carcinogenesis. The following analysis indicates that for some cancers distinct changes in rates during the second and third decades might be associated with the growth and development which occur at puberty.

FIG. 1.—Comparison of growth velocity curves (Tanner *et al.*, 1966) with histograms for bone cancer mortality in England and Wales, 1958–63; males ———, females – – – – –.

TABLE I.—*Modal Age**

| Tissue | Country | Sex | Data: Mortality | Data: Registration |
|---|---|---|---|---|
| Bone | E. & W. | M | 18 | 18 |
| | | F | 16½ | 15 |
| Ovary | E. & W. | F | (18) | (19) |
| | U.S. | F | (19) | — |
| Prostate | E. & W. | M | 18 | — |
| | U.S. | M | 19½ | — |
| Testis | E. & W. | M | 29 | 33 |
| Thyroid | E. & W. | M | — | (28) |
| | E. & W. | F | — | (33) |
| Breast | See de Waard (1969) | | 40–50 | — |

* Parentheses indicate an estimate based on an assumed linear extrapolation.

*Bone Cancer*

It is well known that osteosarcoma, a tumour which occurs mainly in the young, is generally located at sites of maximum growth. Fig. 1 shows a comparison of growth velocities (Tanner, Whitehouse and Takaishi, 1966) with mortality rates for bone cancer (I.C.D. 196)†; the mortality rates were means of annual values for England and Wales during the period 1958–63 (Registrar General, 1958–63). The similarity between the age distribution of the adolescent growth spurt and bone cancer mortality was striking.

For the adolescent growth spurt the maximum growth velocity occurred at 12 years for girls and 14 years for boys (Tanner, Whitehouse and Takaishi, 1966). The estimated modal ages for bone cancer mortality were approximately 4 years later at 18 years for males and 16½ years for females (Table I). The age distribution of bone cancer registrations, available for the period 1962–64 (Registrar General, 1968), was similar to that shown in Fig. 1 for mortality rates. For registrations the estimated modal ages were 18 years for males and 15 years for females (Table I). With an allowance for the time required for symptoms to develop it was clear that changes in bone tumour development followed closely the growth changes at adolescence, with a lag of less than 2 or 3 years.

*Other Cancers*

Because the gonads and other organs also develop rapidly at puberty it was of interest to examine mortality rates for cancer of those organs.

*Ovary*

Average age-specific mortality rates for ovarian cancer (I.C.D. 175) were calculated for England and Wales for the 12-year period 1955–66 (Registrar General, 1955–66). Changes in mortality rate during the second and third decades were not obviously different from changes in later decades.

However, when log (Rate) was plotted against age, rate changes below 30 years could be seen to differ from those above 30 years (Fig. 2). Similar results were obtained for the United States; the rates were mean values for the period

† I.C.D. Seventh Revision.

FIG. 2.—Ovarian cancer mortality in England and Wales (1955–66) and the U.S. (1962–65).

1962–65 calculated from data compiled by Segi and Kurihara (1966, 1969). Log (Rate) for ages above 30 years lay close to a straight line. This line was extrapolated to younger ages and deviations of the observed rate from it assumed to be a measure of those ovarian cancers which were distinct for ages less than 30 years. Modal ages for these deviations were 18 years for England and Wales and 19 years for the United States (Table I). The number of cancers represented by these deviations was approximately one half of the total mortality from ovarian cancers occurring at ages less than 30 years. The precision of this estimate depends of course upon the validity of the assumption of linear extrapolation.

When the analysis was repeated for rates of registration of ovarian cancer (Registrar General, 1968) in England and Wales the modal age was 19 years (Table I).

*Testis*

Mean, age-specific mortality rates for cancer of the testis (I.C.D. 178) were calculated for England and Wales for the period 1955–66 (Registrar General, 1955–66). Rates rose to a maximum at 29 years (Table I). No distinct changes in the rate of increase could be detected for younger ages. Mean registration rates for the period 1962–64 (Registrar General, 1968) had a modal age of 33 years (Table I).

*Prostate*

For the United States and England and Wales the age-specific rate for cancer of the prostate (I.C.D. 177) rose to a peak at the end of the second decade (Table I). The number of cases was, of course, extremely small and the peak during the second decade was of interest only because of its possible association with growth of the prostate at puberty.

*Thyroid*

For ages above 40 years the mean registration rate for thyroid cancer (I.C.D. 194) in England and Wales for the period 1962–64 increased linearly with age (Fig. 3). Below 40 years the rates showed a pronounced peak in the third decade (Fig. 3). When the straight part of the curve was extrapolated to younger ages and subtracted from the recorded rate the differences had a modal age of 28 years for males and 33 for females (Table I).

*Breast*

Breast cancer is extremely rare at ages below 20 years (Close and Maximov, 1965). There is some evidence reviewed recently by de Waard (1969) for a distinct

FIG. 3.—Thyroid cancer registration in England and Wales, 1962–64.

group of breast cancers with a modal age of about 40–50 years. Scrutiny of breast cancer rates did not indicate any earlier accumulation.

### DISCUSSION

The above analysis has indicated that for several tissues the age dependence of cancer rates differed during the second and third decades from the dependence at later ages.

For bone cancer the early rates were strikingly associated with changes in growth velocity at the adolescent growth spurt. Rates rose to a higher value for males, and at an interval of 2 or 3 years later, than for females, resembling closely the characteristics of the adolescent growth spurt. The histogram for females would be consistent with a steeply rising peak with a modal age of 15–16 years (Fig. 1). The association between bone cancer and growth would be more intimate if the adolescent growth spurt were the culmination of processes which increased from birth onwards. This is not unreasonable since the rapid fall of growth velocity after birth could be a decline in the foetal state of activity of tissues, a state which does not appear to extend beyond the second or third year of life (Hubble, 1969).

Skeletal growth is a chondroplasia accompanied by osteogenesis. Chondrosarcoma does not appear until late in life (Sissons, 1958) while osteosarcoma appears earlier and would constitute the majority of bone tumours which were associated with the adolescent growth spurt. It might be therefore that bone cancer development was associated with processes of skeletal maturation (osteogenesis) rather than skeletal enlargement (chondroplasia). Factors associated with skeletal maturation have been summarized recently by Hubble (1969) and include thyroxine, androgens and oestrogen. It would be of interest to establish whether children with osteosarcoma had an advanced or a delayed skeletal maturation and whether any characteristic disturbance of hormone levels could be recognized.

Lee (1961) described a peak in leukaemia rates during the second decade of life and drew attention to the increased rate of bone cancer in males during the same period, mentioning that growth at adolescence might be one of the contributing factors. The present study has shown that the association between bone cancer and growth is too close to be casual. Two features of the leukaemia peak, described by Lee, suggest that this also might be associated with adolescent growth. Firstly, the peak was higher for males than for females and occurred a few years later. Secondly, the increase appeared to be confined to myeloid leukaemia. Furthermore, the leukaemias had an exceptionally brief history suggesting that they had an aetiology distinct from other leukaemias in childhood.

Increased cancer development following growth at adolescence appeared to occur for several other tissues. For ovary and prostate the rate change occurred during the second decade. The accumulation of cancers of the testis and thyroid did not appear until the end of the third decade and if these were to be attributed to adolescent growth the intervening period would need to be about 15 years. It would be of interest to determine whether characteristic patterns of hormone levels could be recognized for patients who developed early tumours of ovary, testis or prostate. This is likely to be more difficult for these tissues because the tumours might secrete hormones themselves. Also, for the ovary and prostate the proposed " early " group accounted for only a very small proportion of the total. The role of pubertal changes could be more effectively studied if the early tumours were of a distinct histological type. For instance dysgerminoma is more frequent during the second decade (Mueller, 1950). Also ovarian sarcoma and solid ovarian carcinoma are relatively more common in the young than older age groups (Huffman, 1968).

The greater incidence of thyroid cancer in women suggests that sex hormones are involved. Rawson and Leeper (1968) have suggested that the presence of sex hormones is associated with benign conditions of thyroid growth. Thus the early group of deaths from thyroid cancer might appear in patients with inadequate levels of sex hormones.

The development of breast cancer is profoundly influenced by child bearing, particularly if this occurs early (see Cole and MacMahon, 1969). Cole and MacMahon (1969) have proposed that the lower risk might be a consequence of the changed hormonal levels during pregnancy. Lilienfeld (1963) suggests that marital status—that is, presumably, child-bearing—influences development of breast cancer late in life. Thus if abnormalities in the development at puberty of production of sex hormones also contributed to development of breast cancer the effect might not appear until late in life. Whether pubertal growth contributes to the group of breast cancers in middle age is a matter, at the moment, for speculation.

The incidence of thyroid cancer following X-irradiation of the thyroid was an order of magnitude higher when the exposure occurred before puberty compared with exposure in adulthood. Hempelmann (1968) has pointed out that, in keeping with a multi-stage theory for carcinogenesis, events initiated by X-rays could be promoted by the normal growth of the thyroid at puberty. This promotion would be absent if exposure took place in adulthood. It is of interest to speculate that exposure of bone, ovary, testis and prostate to carcinogens might give higher yield of cancers if the exposure occurred before puberty. Armitage and Doll (1957) have accounted satisfactorily for age-specific cancer rates throughout adult life by assuming a two-stage mechanism with an intervening exponential growth. This theory might also fit the age distribution of the early cancers discussed in this paper if the exponential term were replaced by a function which described natural growth at puberty.

The author wishes to express his grateful thanks to Miss Alice Duncan for her painstaking technical assistance.

### REFERENCES

Armitage, P. and Doll, R.—(1957) *Br. J. Cancer*, **11**, 161.
Close, M. B. and Maximov, N. G.—(1965) *Archs Surg., Chicago*, **91**, 386.
Cole, P. and MacMahon, B.—(1969) *Lancet*, i, 604.
Hempelmann, L. H.—(1968) 'Thyroid Neoplasia', edited by S. Young and D. C. Inman. New York (Academic Press), p. 267.
Hubble, D.—(1969) ' Paediatric Endocrinology '. Oxford (Blackwell).
Huffman, J. W.—(1968) ' The Gynaecology of Childhood and Adolescence '. Philadelphia (W. B. Saunders, Co.).
Lee, J. A. H.—(1961) *Br. med. J.*, i, 989.
Lilienfeld, A. M.—(1963) *Cancer Res.*, **23**, 1503.
Mueller, C.—(1950) *Am. J. Obstet. Gynec.*, **60**, 153.

RAWSON, R. W. AND LEEPER, R.—(1968) 'Thyroid Neoplasia', edited by S. Young and D. C. Inman. New York (Academic Press), p. 159.

REGISTRAR GENERAL'S Statistical Review of England and Wales (1955–66). London (H.M. Stationery Office).

REGISTRAR GENERAL'S Statistical Review of England and Wales (1968) Supplement Cancer for 1962–64. London (H.M. Stationery Office).

SEGI, M. AND KURIHARA, M.—(1966) 'Cancer Mortality for Selected Sites in 24 Countries', No. 4. Department of Public Health, Tohoku University, Sendai, Japan.—(1969) 'Cancer Mortality for Selected Sites in 24 Countries', No. 5. Department of Public Health, Tohoku University, Sendai, Japan.

SISSONS, H. A.—(1958) 'Cancer', edited by R. W. Raven. London (Butterworth & Co.), Vol. 2, p. 324.

TANNER, J. M., WHITEHOUSE, R. H. AND TAKAISHI, M.—(1966) *Archs Dis. Childh.*, **41**, 454, 613.

DE WAARD, F.—(1969) *Int. J. Cancer*, **4**, 577.